# Evaluation of factors associated with noncompliance in users of combined hormonal contraceptive methods: a cross-sectional study: results from the MIA study

**DOI:** 10.1186/1472-6874-13-38

**Published:** 2013-10-20

**Authors:** Txantón Martínez-Astorquiza-Ortiz de Zarate, Teresa Díaz-Martín, Txantón Martínez-Astorquiza-Corral

**Affiliations:** 1 Department of Gynecology, Hospital de Cruces, 48903 Barakaldo, Spain

**Keywords:** Compliance, Combined oral contraceptive, Transdermal (contraceptive) patch, Vaginal (contraceptive) ring, Switching

## Abstract

**Background:**

Understanding contraception from the perspective of the user may help to improve compliance. The aim of this project was to determine the factors that influence the noncompliance in young women that use combined hormonal contraceptives (pill, patch or vaginal ring).

**Methods:**

A nationwide cross-sectional multicenter epidemiology study. Physicians [obstetricians/gynecologists]) recorded socio-demographic, clinical and current contraception data of 8,762 women. Women completed a self-administered questionnaire on compliance. After the assessment of self-administrated questionnaire, the physicians reported on their recommendations on the possibility of changing the contraceptive.

**Results:**

Fifty-two percent of women were noncompliant, mainly because of simple forgetfulness (pill, 74.9%; patch, 47.8%; vaginal ring, 69.1%). The percentage of noncompliant women was lower in vaginal ring users (26.6%) than in patch users (42.4%) or pill users (65.1%) (p < 0.0001). The most common course of action after noncompliance was to take/use the contraceptive as soon as possible. In the multiple logistic regression analysis, the use of the pill increased the probability of noncompliance compared with the patch and the vaginal ring (odds ratio [IC95%]: 2.53 (2.13-3.02) and 4.17 (3.68-4.73, respectively), and using the patch compared with the vaginal ring (1.65 (1.36-1.99)). Others factors associated with noncompliance were: high treatment duration, low degree of information on the contraceptive method, understanding of instructions on the contraceptive method, indifference to becoming pregnant, lack of partner support, not participation in selecting the method, not having a routine for taking treatment and difficulties remembering use the contraceptive method. Switching contraceptive method was proposed by the physicians to 43.2% of women (51.8% of pill users, 58.2% of patch users and 19.4% of vaginal ring users).

**Conclusions:**

More than 50% of combined hormonal contraceptive users did not comply with the treatment regimen. The percentage of noncompliant women was lower between vaginal ring users. Understanding user’s reasons for noncompliance by the clinician and encouraging a collaborative approach can go a long way to improving compliance.

## Background

4.7 million European women aged 15–49 are estimated to be at risk of an unintended pregnancy [1]. A national survey in Spain showed that, although 69.1% of women of childbearing age used some type of contraception [2], the efficacy of the method was poor in 5% of the cases. In addition, 30.9% of women did not use any method, leaving more than 1 million women at risk of unintended pregnancy. The consequences of noncompliance are far reaching. According to Darney [3], almost half of the 6.3 million pregnancies in the US are unintended, despite the availability of a wide variety of highly effective contraceptive methods.

Combined oral hormonal contraceptives are a safe and efficacious method of protection against unintended pregnancy. However, they are not always used consistently [4], and as many as 47% of women using combined oral contraceptives in the United States miss at least 1 pill per cycle [5]. A European study showed that 19% of women aged 16 to 30 years missed 1 or more pills per cycle and those women were 2.6 times more likely to have an unintended pregnancy than the ones who miss less than 1 pill per cycle [6]. The efficacy of oral contraception depends on continued intake [6]. In many cases, poor compliance can be attributed to human factors [7], such as lack of a fixed routine for pill taking, misinterpretation of the information in the package insert, scant advice from health care providers, and side effects (eg, bleeding irregularities, nausea, and breast tenderness) [6]. Incorrect pill taking can lead to discontinuation and unintended pregnancy [4,8].

Therefore, methods that reduce the frequency of dosing could increase compliance [8]. Alternative delivery methods for combined hormonal contraceptives have been developed over the years; such as the transdermal patch and the vaginal ring [9]. Matters of convenience seem to be especially important for women when selecting the patch or ring. Both offer advantages over the pill; for example, they can be used weekly or monthly, are easy of use and have lower likelihood of forgetting it [10].

Women’s perceptions influence their contraceptive decisions. Indeed, poor compliance generates a significant care burden in terms of user’s anxiety about preventing pregnancy and an increase of visits to the gynecologist for advice [11]. To support informed contraceptive decision-making, healthcare professionals should realize that the perception women have on the method’s ease of use is more important than perceived efficacy, tolerability, health benefits or risks [10].

Acceptance of the vaginal ring has been shown to be higher (46%) than for the pill (39%) and skin patch (15%), mainly because of the lower probability of inadvertent omission [12]. In addition, the efficacy and tolerability profile of the vaginal ring is comparable to that of combined oral contraceptives [13], and women are more likely to continue using it than women who take a combined oral contraceptive [14]. Other authors report the degree of satisfaction with the transdermal patch to be higher than with other delivery methods [15].

Studying the reasons for noncompliance enables us to understand contraception from the point of view of the user, and some authors have assessed the self-reported impact of noncompliance, which may have a negative impact in the user’s behavior in front of working activities and/or with their couple [11].

The primary objective of the study was to determine what factors are associated with noncompliance in two types of user: 1) young women (18–28 years) or 2) women with little experience of combined hormonal contraceptives (maximum 2 years before the visit), in the case of women over 28 years.

The analyzed data about the advice given to users by the gynecologists and the level of acceptance of this advice should place clinicians in a better position to help women select the most suitable method for them or to recommend a more effective method when necessary.

The study describes the profile of the noncompliant user and presents the advice given by the gynecologist depending on the degree of noncompliance, user profile and reasons for noncompliance.

## Methods

In order to analyze compliance with contraceptive use a nationwide cross-sectional multicenter study was performed. Subjects were recruited both in centers of the Spanish National Health Service and in private health centers. Since the primary endpoint of the study was to describe the factors associated with noncompliance and these would only be described by the noncompliant user; the size of the sample must be estimated taking into account the market share in Spain of the various contraceptive methods and the estimated percentage of noncompliant users for each of them (REMO Study: 71% pill, 32% patch and 21.6% vaginal ring) [11]. Based on the estimated market share in Spain of various studies of contraceptive methods (pill 74%, patch 7%, vaginal ring 19%) and an assumption of an overlap of 80% between the 2 types of user (young women and women with little experience of their contraceptive method) a total of 12,000 users was calculated to be necessary to guarantee a minimum sample of 380 per group (pill, patch or vaginal ring) with a margin of error of 5% and a maximum uncertainty in noncompliance (p = q = 0.5), even in the least used method (patch).

In order for women being included in the study they had to be currently using a combined hormonal contraceptive (pill, patch or vaginal ring). In addition, women had to meet at least one of the following inclusion criteria: 1) had to be aged 18 to 28 years or 2) have little experience of their chosen contraceptive method (maximum 2 years before the visit). Women participating in a clinical trial or who were not capable of completing the questionnaire (investigator criteria) were excluded.

Data were collected from the clinical history at a single routine visit to the gynecologist [see Additional file 1]. The physician recorded sociodemographic data (age, educational level, occupational status, stable partner), obstetric history (number of births, abortions [spontaneous and induced], number of live childbirths), and data on the current contraceptive method. A self-administered questionnaire on contraceptive compliance was completed by the woman during the visit. The type of questionnaire varied according to whether they used pill, patch, or vaginal ring. A lack of strict compliance was evaluated by means of questions on forgetting to take the contraceptive, taking the contraceptive late, discontinuing the contraceptive, and incorrect use of the contraceptive. In cases of noncompliance, the woman was asked about the reasons and circumstances that led to noncompliance.

A compliant user was defined as a woman who always used her contraceptive method without no delays or omissions, according to the question in the self-administered questionnaire “Have you delayed/forgotten sometime in the use of your contraceptive method? (Yes/No)”. A noncompliant user was defined as one who missed or delayed using her contraceptive method at least once since the last visit. Delay was defined according to the Summary of Product Characteristics of each method.

Finally, after assessing the self-administered questionnaire, taking into account the user’s profile, the degree of noncompliance and its reasons, the gynecologist recorded his/her recommendation at the visit, as was the woman’s acceptance or rejection of the recommendation and the current type of contraception and the type that was recommended. If a change of method was recommended, the reason for this recommendation was indicated too.

The Ethics Committee of Hospital Clínic (Barcelona, Spain) approved the study according to the legislation in force at the time (Spanish Ministerial Order SAS/3470/2009), and all the patients gave their written informed consent to participate.

The characteristics of the women were described using a frequency table for categorical variables and measures of central tendency and dispersion for continuous variables. A bivariate analysis was performed to describe women’s profile in terms of the contraceptive method used. Contingency tables (of categorical variables) were analyzed using the chi-squared test or Fisher exact test. Continuous variables were analyzed using a *t* test or an analysis of variance, as long as the normality assumption was satisfied; non-normally distributed variables were analyzed using a nonparametric method (Wilcoxon-Mann–Whitney or Kruskal-Wallis) for independent samples. A multiple logistic regression analysis (stepwise method) was performed to determine the factors that affect whether a woman is compliant or not. Statistical significance was set at a 2-sided p-value of < .05. The statistical analysis was performed using SAS v.9.2 (SAS Institute Inc., Cary, North Carolina, USA).

## Results

The gynecologists collected data of 9,367 women, from which 8.762 (93.5%) met inclusion criteria (1. young women [18–28 years] or 2. women over 28 years with little experience of combined hormonal contraceptives (maximum 2 years before the visit)).

Mean (SD) age was 25.3 (4.8) years and most of the women were nulligravid. In addition, 57.3% worked outside the home and 27.2% were students (Table 1). The most commonly used contraceptive method was the pill (61.9%), followed by the vaginal ring (28.4%) and the patch (9.8%). The pill had also been used for a longer period of time than the other methods (mean 30.8 months vs 19.3 and 21.6 months for the patch and vaginal ring, respectively [p < 0.0001]).

**Table 1:** Profile of the study population according to delivery system

	**Total**	**Pill**	**Patch**	**Ring**	**p Value**
Age, years (mean (SD))	25.29 (4.79)	25.14 (4.81)	25.49 (4.78)	25.56 (4.71)	<0.0001^a^
Time using the current method, months (mean (SD))	27.08 (24.84)	30.83 (28.40)	19.28 (15.07)	21.57 (16.01)	<0.0001^a^
Obstetric history (mean (SD))					
Pregnancies	0.36 (0.79)	0.36 (0.79)	0.52 (0.87)	0.32 (0.75)	<0.0001^a^
Deliveries	0.26 (0.60)	0.26 (0.60)	0.37 (0.71)	0.22 (0.56)	<0.0001^a^
Abortions (spontaneous)	0.05 (0.26)	0.05 (0.25)	0.09 (0.35)	0.05 (0.23)	0.001^a^
Abortions (induced)	0.08 (0.35)	0.09 (0.38)	0.12 (0.38)	0.06 (0.27)	<0.0001^a^
Live childbirths	0.17 (0.50)	0.17 (0.50)	0.22 (0.58)	0.14 (0.47)	0.0004^a^
Stable partner					
Yes	7,106 (82.1%)	4,367 (81.5%)	679 (80.6%)	2,060 (83.9%)	<0.0153^b^
No	1,549 (17.9%)	991 (18.5%)	164 (19.5%)	394 (16.1%)	
Education					<0.0001^b^
Primary	934 (10.8%)	655 (12.2%)	109 (12.9%)	170 (6.9%)	
Secondary	3,885 (44.8%)	2,479 (46.2%)	406 (47.9%)	1,000 (40.5%)	
University	3,863 (44.5%)	2,233 (41.6%)	333 (39.3%)	1,297 (52.6%)	
Occupational status					<0.0001^b^
Working outside the home	4,960 (57.3%)	3,018 (56.28%)	476 (56.4%)	1,466 (59.6%)	
Homemaker	561 (6.5%)	363 (6.8%)	90 (10.7%)	108 (4.4%)	
Student	2,353 (27.2%)	1,451 (27.1%)	208 (24.6%)	694 (28.2%)	
Unemployed	790 (9.1%)	530 (9.9%)	70 (8.3%)	190 (7.7%)	
Reasons for omission/delay					
Forgetfulness/delays	2,622 (71.2%)	1,996 (74.9%)	172 (47.8%)	494 (69.1%)	<0.0001^b^
Altered libido	214 (5.8%)	180 (6.8%)	14 (3.9%)	20 (3.0%)	0.0003^b^
Weight gain	211 (5.7%)	201 (7.5%)	4 (1.1%)	6 (0.9%)	<0.0001^b^
Common course of action after noncompliance					
Use the contraceptive as soon as possible	2,396 (65.1%)	1,745 (65.5%)	235 (65.3%)	416 (63.3%)	0.5868 ^b^
Consult a health care provider	598 (22.1%)	337 (16.2%)	76 (14.1%)	145 (21.1%)	<0.0001^b^
Use two or more methods to compensate for the days missed	353 (9.6%)	339 (12.7%)	5 (1.4%)	9 (1.4%)	<0.0001^b^
Routine method					<0.0001^b^
At the same time every day	5,837 (67.6%)	3,621 (67.6)	515 (61.0%)	1,701 (70.0%)	
Daily task	1,472 (17.1%)	933 (17.4)	168 (19.9%)	371 (15.3%)	
Thinking of it but not yet organized	712 (8.3%)	514 (9.6%)	85 (10.1%)	113 (4.7%)	
Not interested	183 (2.1%)	117 (2.2%)	38 (4.5%)	28 (1.2%)	
Others	429 (5.0%)	174 (3.3%)	38 (4.5%)	217 (8.9%)	
Difficulties remembering to use the contraceptive method					
During the week, from Monday to Friday	856 (9.8%)	510 (9.4%)	86 (10.1%)	260 (10.5%)	0.3234 ^b^
On vacation	2,745 (31.3%)	1,531 (28.2%)	279 (32.6%)	935 (37.6%)	<0.0001^b^
On weekend	2,426 (27.7%)	1,889 (34.8%)	175 (20.4%)	362 (14.6%)	<0.0001^b^
On short trips	966 (11.0%)	606 (11.2%)	101 (11.8%)	259 (10.4%)	0.4592 ^b^
Going out the night before	1,127 (12.9%)	863 (15.9%)	114 (13.3%)	150 (6.0%)	<0.0001^b^
Travelling to a different time zone	513 (5.9%)	206 (3.8%)	46 (5.4%)	261 (10.5%)	<0.0001^b^

^a^Kruskal-Wallis test.

^b^Chi-squared test.

p < 0.05.

SD: Standard Deviation.

**Figure 1:**
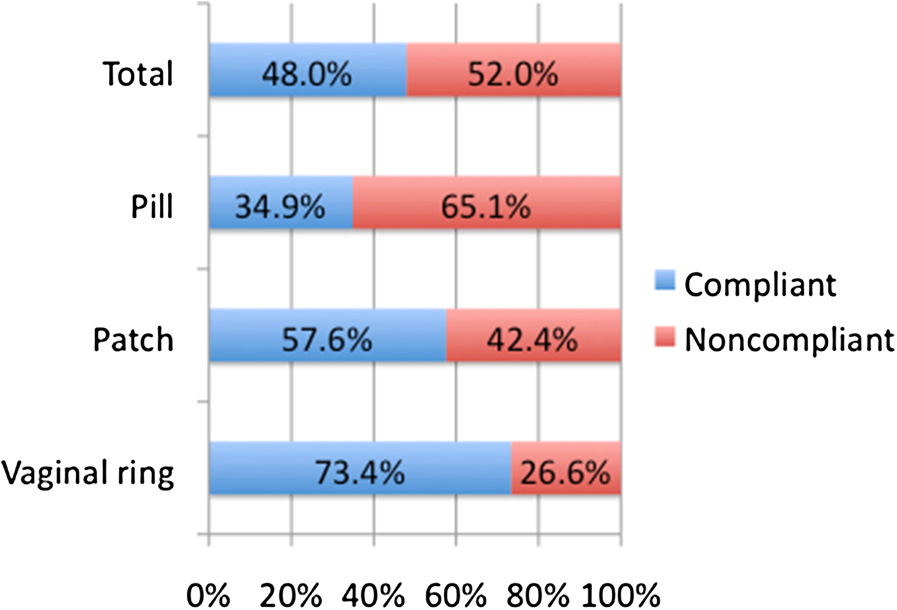
Compliance/noncompliance according to the contraceptive method used.

According to the definitions in the Material and methods section, 52.0% of women were noncompliant, with significant differences depending on the type of contraception used (p < 0.0001) (Figure 1).

The most common reasons for omission/delay of using a contraceptive method were simple forgetfulness (Table 1), with differences observed between contraceptive methods (p < 0.0001). Specific reasons of each contraceptive method included the desire to hasten the occurrence of /postpone withdrawal bleeding (13.6%) and accidental expulsion (6.9%) in women using the vaginal ring. In the case of the patch, the reasons included accidental detachment without the user realizing (29.4%), acne and discomfort when sand got below the patch. In pill users, the reasons included not being in the right place, hastening the occurrence of withdrawal bleeding and rest period.

Most women had a routine to remind them to take/use their contraceptive method, observing statistically significant differences between the contraceptive method used (p < 0.0001). Remembering to use the contraceptive method was more difficult on vacation (31.3%), especially in vaginal ring users (p < 0.0001), during the weekend (27.7%) and after going out the previous night (12.9%), especially in pill users (p < 0.0001). Women also found it difficult to use their contraceptive during trips to different time zone areas (p < 0.0001) (Table 1).

The most common course of action after noncompliance was to use the contraceptive as soon as possible. Women also consulted a health care provider, especially vaginal ring users (p < 0.0001). Almost 12.7% of the women taking the pill used two or more methods to compensate for the days missed compared with 1.4% for women using the vaginal ring (p < 0.0001). The next most common decision was to do nothing, more often between pill users (p < 0.0001).

The compliance comparison showed that the amount of time using the current method (months) was higher in noncompliant women than in compliant ones(24.8 compliant women vs 29,1 noncompliant women; p < 0.0001), and that the degree of information and degree of understanding the information and instructions of the used method were lower in noncompliant women compared to compliant women. More than half of the women (51.1%) believe that if they could participate in the choice of the contraceptive method they would have less forgetfulness/delays, mainly noncompliant women compared with compliant women (57.0% vs 44.1%, p <0.0001). The partner support and interest in not becoming pregnant were also lower in noncompliant women. Not having a routine or having difficulties remembering to use the contraceptive method involves higher noncompliance (Table 2).

**Table 2:** Profile of the study population according to compliance

	**Total**	**Compliant**	**Noncompliant**	**p Value**
Age, years (mean (SD))	25.29 (4.79)	25.50 (4.77)	25.10 (4.79)	<0.0001^a^
Time using the current method, months (mean (SD))	27.08 (24.84)	24.82 (23.28)	29.16 (26.04)	<0.0001^a^
Obstetric history (mean (SD))				
Pregnancies	0.36 (0.79)	0.35 (0.74)	0.38 (0.83)	<0.3966^a^
Deliveries	0.26 (0.60)	0.25 (0.57)	0.27 (0.63)	<0.5212^a^
Abortions (spontaneous)	0.05 (0.26)	0.05 (0.26)	0.05 (0.26)	<0.7648^a^
Abortions (induced)	0.08 (0.35)	0.06 (0.27)	0.10 (0.42)	<0.0001^a^
Live childbirths	0.17 (0.50)	0.15 (0.47)	0.18 (0.53)	0.0436^a^
Stable partner				<0.0001^b^
Yes	7,106 (82.1%)	3,525 (84.9%)	3,581 (79.5%)	
No	1,549 (17.9%)	628 (15.1%)	921 (20.5%)	
Education				<0.0001^c^
Primary	934 (10.8%)	358 (8.6%)	576 (12.8%)	
Secondary	3,885 (44.8%)	1,817 (43.6%)	2,068 (45.8%)	
University	3,863 (44.5%)	1,993 (47.8%)	1,870 (41.4%)	
Occupational status				<0.0003^c^
Working outside the home	4,960 (57.3%)	2,474 (59.5%)	2,486 (55.2%)	
Homemaker	561 (6.5%)	244 (5.9%)	317 (7.0%)	
Student	2,353 (27.2%)	1,091 (26.2%)	1,262 (28.0%)	
Unemployed	790 (9.1%)	349 (8.4%)	441 (9.8%)	
Information on treatment				<0.0001^c^
Very informed	902 (44.8%)	2,309 (55.2%)	1,593 (35.1%)	
Normal	4,495 (51.5%)	1,814 (43.4%)	2,681 (59.2%)	
Not very informed	307 (3.5%)	61 (1.4%)	246 (5.4%)	
Not interested	15 (0.2%)	0 (0%)	15 (0.3%)	
Understanding of information and instructions				<00001^c^
Not interested	11 (0.1%)	2 (0.05%)	9 (0.2%)	
Anything	20 (0.2%)	4 (0.1%)	16 (0.4%)	
Not understanding a lot of things	393 (4.5%)	70 (1.7%)	323 (7.1%)	
Understanding almost all	2,719 (31.2%)	1,046 (24.9%)	1,673 (36.9%)	
Complete understanding	5,587 (64.1%)	3,076 (73.3%)	2,511 (55.4%)	
Partner support				<.0001^c^
Always reminding	2,992 (34.3%)	1,724 (41.2%)	1,268 (28.1%)	
Absence of partner	1,128 (13.0%)	458 (10.9%)	670 (14.8%)	
Indifferent	1,349 (15.5%)	540 (12.9%)	809 (17.9%)	
Sometimes	3,239 (37.2%)	1,467 (35.0%	1,772 (39.2%)	
Interest in not becoming pregnant				<.0001^c^
Very interested	5,823 (66.7%)	2,893 (68.9%)	2,930 (64.6%)	
Interested	1,551 (17.8%)	737 (17.6%)	814 (18.0%)	
Prefer not to get pregnant	1,174 (13.5%)	489 (11.7%)	685 (15.1%)	
Not mind	179 (2.0%)	76 (1.8%)	103 (2.3%)	
Belief that participation would avoid forgetfulness/delays				<0.0001^c^
More	564 (6.6%)	349 (8.6%)	215 (4.8%)	
The same	3,613 (42.3%)	1,905 (46.9%)	1,708 (38.2%)	
Less	4,357 (51.1%)	1,808 (44.5%)	2,549 (57.0%)	
Routine method				
At the same time every day	5,837 (67.6%)	2,990 (72.1%)	2,847 (63.5%)	< 0.0001^c^
Daily task	1,472 (17.1%)	746 (18.0%)	726 (16.2%)	
Thinking of it but not yet organized	712 (8.3%)	96 (2.3%)	616 (13.7%)	
Not interested	183 (2.1%)	60 (1.5%)	123 (2.7%)	
Other	429 (4.3%)	254 (6.1%)	175 (3.9%)	
Difficulties remembering to take/use/remove the contraceptive method				
During the week, from Monday to Friday	856 (9.8%)	221 (5.3%)	635 (14.0%)	< 0.0001^b^
On vacation	2,745 (31.3%)	1,372 (32.6%)	1,373 (30.2%)	0.015 ^b^
On weekend	2,426 (27.7%)	756 (18.0%)	1,670 (36.7%)	< 0.0001^b^
On short trips	966 (11.0%)	492 (11.7%)	474 (10.4%)	0.0604^b^
Going out the night before	1127 (12.9%)	422 (10.0%)	705 (15.5%)	< 0.0001^b^
Travelling to a different time zone	513 (5.9%)	328 (7.8%)	185 (4.1%)	< 0.0001^b^

^a^Wilcoxon-Mann–Whitney test.

^b^Fisher exact test.

^c^Chi-squared test.

p < 0.05.

SD: Standard Deviation.

A multiple logistic regression analysis (stepwise method) was performed to determinate which factors might affect the probability of noncompliance of a user (Table 3). The analysis revealed that the length of time using the contraceptive method was likely to play a role in the noncompliance therapy [OR (95% CI): 1.006 (1.004-1.008), p < 0.0001]. Depending on the contraceptive method the level of noncompliance can be higher (p < 0.0001): using pill increased the probability of noncompliance compared with the patch [OR (95% CI): 2.53 (2.13-3.02)] and compared with the vaginal ring [OR (95% CI) 4.18 (3.68-4.73)], and the use of patch increased the probability of noncompliance [OR (95% CI): 1.65 (1.36-1.99)] compared with the vaginal ring. Low level of user’s information treatment and a poor understanding of information and instructions were also associated with a higher probability of noncompliance. Compared with women interested not becoming pregnant, the probabilities of noncompliance increased for women preferring to not get pregnant [OR (95%): 1.27 (1.06-1.53)] and women who would not mind [OR (95%): 1.46 (1.00-2.12)]. Having a partner indifferent to a possible pregnancy or the absence of a partner also increased the probability of noncompliance. Moreover, the lack of support from one’s partner or the absence of a partner was also a factor that may affect the noncompliance increased. The probability of noncompliance was higher in women who think that if they could participate in the decision to choose the contraceptive method would have fewer delays/omissions, that it, they would be more compliant.

**Table 3:** Multiple logistic regression analysis

**Factor**		**OR estimate**	**OR 95% CI**	**p-value**
Time using the current method, months (continuous variable)		1.006	1.004–1.008	<0.0001
Contraceptive method	Pill vs vaginal ring	4.175	3.683–4.732	<0.0001
	Patch vs vaginal ring	1.648	1.362–1.994	
	Pill vs Patch	2.534	2.128–3.016	
Information on treatment	Not very informed	1.931	1.336–2.791	<0.0001
(reference: very informed)	Normal	1.532	1.370–1.713	
Interest in not becoming pregnant	Very interested	1.161	1.012–1.332	<0.0001
(reference: interested)	Prefer not to get pregnant	1.273	1.057–1.534	
	Not mind	1.455	1.001–2.117	
Partner support	Absence of partner	1.415	1.195–1.676	<0.0001
(reference: always reminding)	Indifferent	1.416	1.205–1.664	
	Sometimes	1.372	1.214–1.550	
Understanding of information and instructions	Not interested	2.137	0.182–25.052	0.0089
(reference: complete understanding)	Anything	1.066	0.283–4.014	
	Not understanding a lot of things	1.696	1.213–2.371	
	Understanding almost all	1.167	1.032–1.318	
Belief that participation would avoid forgetfulness/delays	More *vs* The same	1.072	0.865–1.329	<0.0001
	Less *vs* The same	1.298	1.167–1.444	
	Less *vs* More	1.211	0.977–1.501	
Routine method	Other	1.142	0.902–1.447	<0.0001
(reference: at the same hour each day of the week/month)	Not interested	1.470	1.003–2.154	
	Considered but not organized	3.873	2.997–5.004	
	Activity reminding treatment	0.846	0.740–0.968	
Difficulties remembering to take/use/remove the contraceptive method	During the week, from Monday to Friday	6.189	4.982–7.687	<0.0001
(reference: none)	On vacation	2.132	1.851–2.455	<0.0001
	On weekend	3.392	2.934–3.921	<0.0001
	On short trips	1.563	1.306–1.871	<0.0001
	Going out the night before	2.099	1.762–2.501	<0.0001
	Travelling to a different time zone	1.403	1.106–1.780	<0.0001

Goodness-of-fit (Hosmer-Lemeshow): p-value = 0.7004.

Discrimination test: p-value = 0.7830.

*Abbreviations*: *CI* Confidence Interval.

Probability modeled is noncompliance (code = 1).

The use of a method to remember the medication was another factor associated with the noncompliance. Not being interested in a routine method, having considered it but not being organized yet and doing an activity that provided a reminder on treatment increased the noncompliance compared always taking or applying the medication always at the same hour.

Finally, the noncompliance increased in women with difficulties remembering to take/use/remove the contraceptive method: during the week (Monday-Friday), on weekends, on vacation, on short trips, after going out the night before and when travelling to a different time zone).

A recommendation for switching was made regardless of the current method (43.2%), although this recommendation was made to a lower percentage of women using the vaginal ring than to the other groups (pill, 51.8%; patch, 58.2%; vaginal ring, 19.4%). The reasons given by the gynecologist for switching method varied according to the current method and the degree of compliance. Patients were advised to switch to the pill for reasons of tolerance. In the case of the patch or vaginal ring, the switch was recommended for reasons of convenience in compliers and for reasons of compliance in noncompliers.

## Discussion

The study analyzed 3 widely used contraceptive methods in a sample that was sufficiently large to enable to present the results with a significant degree of precision (the precision reached with the final sample was 1%. It is not significantly difference from the initially proposed sample -0.9%-). Most of the women in the study used the pill (61.9%), a finding that is consistent with the results of a survey of contraceptive use among women from 5 European countries [1].

Compliance varies according to the delivery method chosen. That the study showed that more than half (52.0%) of the women do not apply their contraceptive method according to the instructions in the package insert, especially in pill users. The main reason for missing/delays in using the contraceptive method in the study was simple forgetfulness, although some women complained of side effects such as altered libido or weight gain. A large noncompliance rate and women's difficulties in maintaining safe contraception after missing a pill were observed in others studies [16,17]. Westhoff et al. [18] found that most women discontinued for reasons not associated with side effects. One study showed that compliance was greater with the vaginal ring than with the pill [19], whereas others show consistent perfect compliance with the transdermal patch [20,21].

Not having a regular routine has been shown to be one of the strongest risk factors for poor compliance. Women who do not have a regular routine for pill taking are more than 3 times more likely to be inconsistent users [6]. Memory aids such as associating pill taking with another daily action or leaving the pill package visible, have also been associated with easier compliance [22].

The study shows that not having a stable partner increased noncompliance. Whenever both members of a couple are convinced that they do not want a pregnancy, the participation of the partner has been shown to be effective in increasing adherence [23]. This study, in agreement with other studies, also demonstrated that the low level of information on the contraceptive treatment and poor understanding of information and instruction influence noncompliance [24].

Indeed, several authors have shown that the quality of family planning counseling could affect continuation with a particular method [25]. Halpern et al. [7] suggested that, while enhanced counseling has a limited effect on continuation, it could change the reasons why women discontinue contraception. Furthermore, Grove and Hooper [26] stress the need for physicians to make recommendations that are tailored to a woman’s medical profile and preferences.

The relationship with the health care provider is paramount in ensuring compliance, and one study of adolescent women showed that if the physician was considered to be helpful, then users were more compliant [27].

In addition, the physician must be able to provide an alternative method, should compliance prove difficult with the initially chosen one [28]. In this study, a recommendation for switching was made by the clinician after analyzing the reasons for noncompliance of the users, although this recommendation was made to a lower percentage of women using the vaginal ring than to the other groups (pill, 51.8%; patch, 58.2%; vaginal ring, 19.4%).

This study draws the attention to the gynecologist about the factors associated with noncompliance to the contraceptive method (pill, patch, vaginal ring), so the health professional can recommend a change of treatment that best suits the user profile.

Nevertheless, the study is subject to a series of limitations. First its cross-sectional design means that it is not possible to describe those factors that affect compliance; making unable to predict outcomes or draw conclusions about causal relationships. Second, as the study population comprises young women who may have little experience of using combined hormonal contraceptives, our findings cannot be extrapolated to other age groups. Third, a more accurate definition of compliance would enable us to rule out any potential bias in the comparison of the 3 methods. Fourth, the used method of assessing compliance was indirect, namely, by self-administered questionnaire; therefore the resulting data are not as objective as those obtained using more direct methods.

## Conclusions

The findings from this study conclude that more than half of the women were noncompliant, but the frequency of their noncompliance varied with the method used. Other factors associated to increase the noncompliance were: high treatment duration, low level information treatment, poor understanding of information and instructions, not interest becoming pregnant, low partner’s support or absence of partner, non-participation in the choice of contraceptive method, low level of interest in a routine method or difficulties remembering to take/use the contraceptive method. Understanding the user’s reasons for noncompliance by the clinician and encouraging a collaborative approach can go a long way to improving compliance.

## Abbreviations

CI: Confidence interval; OR: Odds ratio; SD: Standard deviation.

## Competing interest

The authors declare that they have no competing interests.

## Authors’ contributions

TMAOZ participated in study design, data analysis and interpretation, and drafting and writing the manuscript. TDM and TMAC participated in its design and coordination and helped to draft the manuscript. All authors read and approved the final manuscript.

## Pre-publication history

The pre-publication history for this paper can be accessed here:

http://www.biomedcentral.com/1472-6874/13/38/prepub

## Supplementary Material

Additional file 1 Investigators of the MIA study. Click here for file
