# Supplementary material for: Evaluation of factors associated with noncompliance in users of combined hormonal contraceptive methods: a cross-sectional study: results from the MIA study
Source: BMC Womens Health. 2013 Oct 20;13:38. doi: 10.1186/1472-6874-13-38 (PMC4015733; doi:10.1186/1472-6874-13-38)
Supplement: Additional file 1 — Investigators of the MIA study. [file 1472-6874-13-38-S1.docx]

**Additional File 1: Investigators of the MIA study:**

Mª Luz Lamelas Suarez Poca; Siham Ali Yassin; Edit Lopez-Grado Nerin; Silvia Atan Sande; Montserrat Alcalde Royo; Orlando Saporiti; Tomasz Teziersui; Mª Jose Ortega Jimenez; Javier Haya Palazuelos; Raquel Carracedo Reboredo; Jose Moreno Ruiz; Antonio Sanchez Muñoz; Rosa Mª Huguet Monfulleda; Mar Cadiñanos Diaz-Tejeiro; Ivan Jose Arqueros Alvarez; Meritxell Gispert Gibbayl; Beatriz Perez Gonzalez; Silvana Bonino Alvarez; Sofia Gramatovich Chichek; Sonsoles Andreu Alvarez; Inmaculada Orensanz Fernandez; Juan Trujillo Cuenca; Concepción Martin Perpiñan; Yvonne Cabrera Rodriguez; Francisco Estaca Domingo; Teresa Morante Arias; Antonio Caparros Zubieta; Mª Antonia Martinez Martin; Rafaela Pilar Cruz Santana; Ernesto Vizcaino Argote; Juan Fernandez Rodriguez; Pablo Vallaure Lillo; Luis Miguel Rivera Vela; Gabriela Del Val Garrido; Jose Maroto Gordo-Pacheco; Carmen Oteo Dominguez; Victoria Orallo Toural; Maroun Chebair Charbel; Angelina Gonzalez Alfonso; Marisa Alija Castrillo; Eduardo Yañez Gonzalez; Carmen Celina Garcia Ferreiro; Manuel Guereñu Carnevali; Isidro Jose Lago Barreiro; Isabel Pardiñas Costas; Emilio Cabo Silva; Isabel Sanchez Buitrago; Ana Martinez Gonzalez; Jose Rufino Mendez Arrojo; Yolanda Alonso Castañon; Ignacio Arnott Fernandez; Aurora Lemos Valdes; Rafael Maroto Paez; Alfredo Urrutia Pintado; Marina Barrios Alvarez; Noelia Fernandez Aller; Laura Prieto Sanchez; Beatriz Perez Prieto Mar Garcia Sanchez; Walter Di Mizio Giovannelli; Armand Marti Laporte; Merce Feriche Adell; Anna Chassignet Martin; Rocio Garcia-Moreno Marchan; Beatriz Suarez Alvarez; Jorge Gayete Martinez; Encarnación Laforet Martínez; Genoveva Corral De La Cruz; Jose Diaz Sanchez; Jose Manuel Sande Rodriguez; Javier Blazquez Angulo; Luis Miguel Gonzalez Sejas; Concepción Hernandez Frias; Mª Luisa Cendon Otero; Manuela Amoros Moreno; Alexandra Segura Fernandez; Elisabet Forroll Turro; Narcis Ros Campas; Isabel Rodriguez Bertos; Sandra Persico Costamagna; Ramon Monguio Garcia; Mª Teresa Codina Nesple; Ana Esther Del Villar Vazqiez; Carmen Aceituno Diestro; Fernando Valenzuela Vialardi; Hildegard Mausbach Reisen; Antonio Navarro Sanchez; Salvador Torra Sole; Elisa Laso Perez; Fernando Puig Muñoz; Eva Fresnadillo Humet; Manuel Del Campo Rodriguez; Nuria Granollers Bohils; Jose Ramon Prado Guillen; Laura Forcen Acebal; Lourdes Rodriguez Vilches; Marta Suarez Rodriguez; Isabel Ramirez De La Piscina; Silvia Pilar Gonzalez Rodriguez; Javier Martin Escanciano; Gustavo Padilla Gonzalez; Juan Eloy Asenjo De La Fuente; Rolando Ledea Vazquez; Miquel Montalvo Ibarra; Mª Luisa Fernandez Perez; Eva Sarda Barea; Ana Gonzalez Lopez; Paula Buelga Lopez; Mª Carolina Paladino Decile; Mª Cristina Gonzalez Macho; Carmen Pingarron Santofimia; Pablo De La Fuente Sanz; Antonio Luis Navarro Soler; Jose Francisco Aguirre Ocaña; Silvia Poyo Torcal; Eduardo Adrian Cubillo Rodriguez; Mª Ángeles Prieto Matas; Mirruan Yordi Yordi; Mª Jose Carballo Martinez; Jose Mª Rios Torre; Rafael Fernandez Alvarez; Gloria Angela Maroto Busto; Maria Josefa Medina Bravo; Zakieh Diab Farah; Carme Castells Raurell; Leopoldo Abarca Martinez; Fernando Rodriguez Vila; Maria Carmen Gutierrez Velez; Juana Gallo Del Valle; Paula Suarez Mansilla; Jose Melendez Encalada; Raquel Cajal Lostao; Teresa Diaz Martin; Paloma Pino Villalba; Eugenia Mª Luisa Megias Martin; Cristina De Urzaiz Paccaud; Diana Sojo Guttero; Mª Mercedes Perez Gonzalez; Carmen Dapena Garcia; Olga Ruiz Jimenez; Fernanda Pla Centelles; Montserrat Garcia Ardit; Aurora Francesch; Teresa Sanchez Escofet; Mª Teresa Rodriguez Bengoa; Pablo Iglesias Sobrado; Tomas Cortadella Rosel; Ana Purificación Lopez Roca; Hanna Mohamad Darwich; Pere Carreras Almirall; Juan Arce Vidal; Carme Guasch Carbonell; Emili Muñoz-Ramos Tora; Carme Castells Raurell; Mª Jesus Rodriguez Domingo; Miguel Rejas Gutierrez; Mª Dolors Jose Comi; Ignacia Maria Soroa Mendizabal; Mª Belen Cabezas Sanchez; Juana Garcia Contreras; Carmen Troncoso Miranda; Dolores Reina Lopez; Manuel Garcia Hernandez; Cristina Porras Hidalgo; Sylvia De Los Reyes Peña; Mª Victoria Garcia Pastor; Mª Isabel Mudarra Barrero; Estrella Garcia Piñero; Rosana Parisi Tortorelli; Juana Marta Lencina Lencina; Mª Carmen Machuca Carrilero; Mª Eugenia Leon Carvallo; Mª Jesus Monfort Monfort; Julia Canals Pelaez; Federico Villagrasa Rocher; Clara Julieta Tejera Palacios; Mariano Sanmartin Agullo; Presentación Garcia Martinez; Thaimi Tamayo Ferriol; Isabel Garrido De Orte; Isabel Maria Silva Reus; Leticia Lazzaletta; Nelson Silvino Pio Avella; Catalina Maria Roig Julia; Magdalena Santandreu Torres; Miguel Angel Terrasa Matas; Belén Martinez-Gijon Machuca; Mª Victoria Bartolome De Miguel; Marta Coloma Escribano; Juan De La Cruz Ferrando Garcia; Ibrahim Hachem Jomaah; Antonio Muñoz Garcia; Juan Jose Rascon Poza; Gloria Marchal Corrales; Ivan Gimenez Peralta; Carlos Javier Maroto Diaz; Elena Laynez Herrero; Fernando Marin Rodriguez; Monica Gomez Cortes; Monica Navarro Rodriguez; Matilde Gomez Frieiro; Francisco Fernandez Robayna; Hortensia Garcia Robayna; Mª Beatriz Llop Ventureira; Africa Rebollo Cuadro; Manuel Prados Alonso; Eva Maria Contreras Ariza; Mª Felisa Cadiz Marin; Emilio Lopez Perez; Ángela Escobar Casas; Jose Subiris Gonzalez; Mª Isabel Aguilera Villegas; Francisco Javier Vico De Miguel; Antonio Jesus Fernandez Montes; Manuel Lupiani Gimenez; Juan Ramon Dominguez Hierro; Mª Rosa Sainz Martinez; Amalia Moreno Fraile; Francisca Gomez Molina; Francisco Carlos Zorrilla Romera; Manuel Muñoz Suarez; Mª Teresa Herrera Muñoz; Isabel Mª Leon Doblas; Javier Pantoja Rosso; Mª Antonia Torrejon Eduardo; Mercedes Sainz Hernandez; Alfredo Otalora De La Serna; Mikel Gorostidi Pulgar; Teodora Alonso Gutierrez; Federico Muñoz Martinez De Salinas; Juana Hernandez Hernandez; Ana Mª Ezquerro Ezquerro; Mª Teresa Peinado Rodriguez; Mª Begoña Romo Herrero; Mº Josefa Belar Ortega; Francisco Valle Vicente; Pedro Diez Bueno; Mª Concepción Gallardo Casado; Azucena Llopis Perez; Isabel Duarte Gonzalez; Rosa Maria Del Rey Lobo; Susana Escuder Garbajosa; Carmen Salvador Ballada; Dominica Nieto Bragado; Mª Carmen Pertusa Romero; Mª Concepción Martin Matito; Begoña Alday Asua; Ramon Santana Garcia; Ibis Lami Jimenez; Jorge Ignacio Vaquero Lena; Mª Victoria Bravo Violeta; Nuria Sanchez Perez; Jose Luis Neyro Bilbao; Francisco Javier Lopez Moreno; Miguel Angel Elorriaga Garcia; Nerea Marin Lacarta; Arantza Amondarain Lasa; Amparo Esquiroz Lauroba; Matxalen De La Rica Bilbao; Mª Jesus Rui-Wamba Gonzalez; Pablo Juaristi Oria; Jenaro Oraa Zubeldia; Batirtze Aurrecoechea Uria; Jesus Echevarria Barenechea; Silvia Solaetxe Rodriguez; Ramon Ayllon Martiarena; Jose Angel Navarrina Martinez; Javier Herrera Gimenez; Mª Jose Asunción Ortiza; Jose Javier Griño Andres; Salud Andreu Llacer; Marian Obiol Saiz; Jorge Perpiña Cano; Jose Antonio Canovas Garcia; Mª Dolores Escribano Canovas; Mª Jose Segura Garcia; Ana Mª Gomez La Encina; Antonio Perez Carrion; Mª Isabel Sanchez Zapata; Luis San Juan Rodriguez; Victoria Prada Cimarro; Adela Vidal Chornet; Amparo Prades Sanchis; Consuelo Santamaria Luna; Joaquim Palmer Blanch; Encarnación Lerma Beso; Carmen Castro Diez; Vicente Fuster Cortina; Jorge Iranzo Sola; Ana Fernandez Sainz; Elena Bescos Santona; Mª Concepción Blasco Gimeno; Magdalena Balaguer Fabuel; Jose Vicente Silvestre Visa; Joaquín Garcia Cerveña; Mª Ángeles Maiques Miralles; Concepción Leal Cariñena; Arturo Gil Bretones; Natalia Ibañez Meca; Jose Perez Garcilaso; José Luis Valle Martín; Jose Maria Garcia Loscos; Rita Maiques Diaz; Agustina Melia Palomares; Teresa Aznar Altaba; Antonio Adiego Sancho; Mª Ángeles Vazquez Lopez; Mª Consuelo Muxi Moner; Gloria Rodea Gaspar; Pau Carabias; Jose Antonio Navas Exposito; Elena Ruiz Domingo; Pilar Blanco Acosta; Jose Antonio Iparraguirre Rioja; Maria Gonzalez Rodriguez; Mª Isabel Talens Cerrello; Manuel Romero Cardiel; Monica Tamara Franco Fraiz; M. Consuelo Re Meijide; Matilde Zamorano Delrio; Lidia Mª Varela Gonzalez; Mª Del Mar Agapito Duran; Arantza Meabe Elorza; Mª Mercedes Ley Martos; Mª Begoña Cid Prol; Juan Manuel Cumbraos Alvarez; F Javier Marqueta Sobrino.
